# (MARGOT) Monocular Camera-Based Robot Grasping Strategy for Metallic Objects

**DOI:** 10.3390/s23115344

**Published:** 2023-06-05

**Authors:** Carlos Veiga Almagro, Renato Andrés Muñoz Orrego, Álvaro García González, Eloise Matheson, Raúl Marín Prades, Mario Di Castro, Manuel Ferre Pérez

**Affiliations:** 1 BE-CEM Beams Department, Controls, Electronics and Mechatronics Group, European Organization for Nuclear Research (CERN), 1217 Geneva, Switzerland; 2 Interactive Robotic Systems Lab, Jaume I University of Castellón, 12006 Castellón de la Plana, Spain; 3 Centro de Automatica y Robotica (CAR) UPM-CSIC, Universidad Politecnica de Madrid, 28006 Madrid, Spain

**Keywords:** computer vision, telerobotics, grasping determination

## Abstract

Robotic handling of objects is not always a trivial assignment, even in teleoperation where, in most cases, this might lead to stressful labor for operators. To reduce the task difficulty, supervised motions could be performed in safe scenarios to reduce the workload in these non-critical steps by using machine learning and computer vision techniques. This paper describes a novel grasping strategy based on a groundbreaking geometrical analysis which extracts diametrically opposite points taking into account surface smoothing (even those target objects that might conform highly complex shapes) to guarantee the uniformity of the grasping. It uses a monocular camera, as we are often facing space restrictions that generate the need to use laparoscopic cameras integrated in the tools, to recognize and isolate targets from the background, estimating their spatial coordinates and providing the best possible stable grasping points for both feature and featureless objects. It copes with reflections and shadows produced by light sources (which require extra effort to extract their geometrical properties) in unstructured facilities such as nuclear power plants or particle accelerators on scientific equipment. Based on the experimental results, utilizing a specialized dataset improved the detection of metallic objects in low-contrast environments, resulting in the successful application of the algorithm with error rates in the scale of millimeters in the majority of repeatability and accuracy tests.

## 1. Introduction

Particle acceleration complexes and nuclear plants require continuous maintenance that ideally should be performed remotely due to the presence of radiation, magnetic fields, or lack of oxygen. These health risks limit access to the personnel in charge of service and maintenance activities in the target area. Remote operations become an obligation when a catastrophe occurs, such as in Fukushima [1], where the dispatching of mobile manipulators became a necessity (that generates additional efforts to ensure safety). This requires expert operators for both the teleoperation of robots, as well as the target area, as despite significant improvements, autonomous behavior of robots is difficult to achieve due to the unstructured nature of the environment [2]. At best, where the task is highly repetitive (e.g., obstacle avoidance, distance estimation), the operator might remain in the control loop in a supervised manner with the aim of guaranteeing the security of the performance, otherwise the operator must carry out the whole intervention manually by deploying their skills and experience.

For many such interventions, one of the requirements is the use of a single camera, with the aim of saving space, which is the biggest constraint in terms of hardware due to the nature of the facilities where the interventions must be performed. These cameras can be used to estimate the distance to various objects of interest. Other sensors that can be used for this, e.g., Time of Flight (ToF) sensors, do not always guarantee the success of the measurements on materials that have reflective surfaces [3].

However, the main challenge to be faced is that of dealing with purely metallic targets, devoid of any contrast between them and the surrounding environment. These were not designed for the purpose of remote maintenance, and far less by robots through computer vision techniques, so they have never been endowed with textures that might render them easier to identify and manipulate.

This paper presents a novel system to estimate stable grasping points in metallic targets with a lack of contrast between the object and its environment, regardless of the complexity of its surface, since it searches for the surface flat enough to guarantee the stability of such points. To this end, the problem is divided into three distinct sections:

- Object detection by means of a deep learning architecture specifically designed for salient object detection.
- Determination of the proper stable grasping points by calculating the geometrical and physical properties of the object contour.
- Spatial coordinates estimation for previously estimated grasping points using stereo vision approximation with a monocular system, through the translation of the camera across a specified baseline.

The combination of these three modules generates a robust system able to detect objects in unstructured environments with a lack of features and determine their ideal grasping points according to the available hardware (two-finger gripper) and their spatial position both for an autonomous approach and for teleoperation guidance. The hardware limitations are inherent in the nature of the interventions, where the dimensional boundaries often make the target area too narrow to choose the ideal hardware. This restricts the possibilities and compels researchers to seek a universal solution that can satisfy any requirement at the risk of compromising both the degree of sensitivity and the level of dexterity when executing the task. For these reasons, grippers such as the three-finger gripper one from Kinova^®^ (Kinova Robotics, Boisbriand, QC, Canada), or the angular gripper one from Robotiq^®^ (Levis, QC, Canada) have been discarded for this project.

## 2. State of the Art

Since the beginning, humans have built tools to help them perform tasks, which is why manipulation, and grasping more specifically, is one of the larger fields of research in robotics. Robotic manipulators can be considered as such helpful tools, either when they are operated autonomously in industry [4] or via teleoperation as early as the mechanical Master–Slave Manipulator Mk. 8 (MSM-8) [Central Research Laboratories, Red Wing, MN, USA, 1945]. This project is focused on using robots as tools via teleoperation.

**Figure 1:**
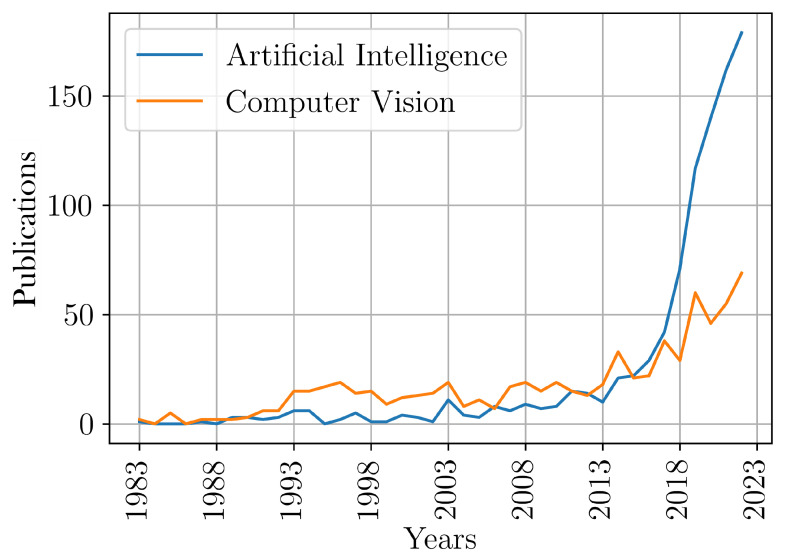
Trend for “Robotics, Grasping, and Computer Vision or Artificial Intelligence” in the last 40 years.

As can be seen in Figure 1, over the last 4 decades more than 90 computer vision studies became the basis for robotic grasping research, which has rapidly evolved towards the use of Artificial Intelligence (AI) during the last decade. These non-exhaustive numbers have been found by analyzing trends in publications dealing with robotics, grasping, and computer vision or Artificial Intelligence from IEEE Xplore and Scopus.

Traditional computer vision methods such as background removal [5], object contour estimation using Histograms of Oriented Gradients (HOG) [6] within a scene, feature extraction techniques including the Scale Invariant Feature Transform (SIFT) [7], or Speeded Up Robust Feature (SURF) [8] have demonstrated limitations in treating complex scenes, where poor contrast, lack of features, or variations in lighting challenge the algorithm’s performance. On the other hand, the AI techniques used for object localization and classification encompass both Machine Learning (ML) and Deep Learning (DL) methods, in which the former comprises of probabilistic approaches such as Bayesian classifiers [9], k-Nearest Neighbor [10], Support Vector Machine [11], a mixture of some of these techniques as shown in [12], or ontology-based techniques [13]. Deep learning (DL) approaches encompass various object detection techniques, including two-stage detectors including R-CNN [14], Faster-RCNN [15], and Mask-RCNN [16], as well as one-stage detectors such as YOLO [17], which is widely utilized for real-time object detection, SSD [18], and RetinaNet [19]. Additionally, semantic segmentation methods including DeepLab V3 [20] and U-Net [21] are also part of DL techniques. However, it is important to note that DL methods necessitate substantial quantities of labeled data for effective training.

Numerous methods have been deployed to determine the grasping point of objects, making use of visual information from single cameras to locate such points, either by edges [22] or image moments [23]. Physics-based grasping uses the physical properties of both the object and the end-effector to estimate its proper grasping points [24,25]. The techniques of using analytical models and geometric algorithms such as force–displacement [26], grasping taxonomy [27], or prediction of forces on living objects [25], as well as a combination of multiple methods [28], perform well as long as the targets do not have a complex or irregular geometry. Some methods have been recently developed, such as [29,30], which make use of deep neural networks, or the combination of information from several sensors for multi-modal grasping as in [31,32]. Although both solutions present a high level of robustness and accuracy, they require a very large data set.

Reinforcement Learning (RL) brings a new strategy by letting an agent learn while receiving rewards for fulfilling assignments, thus teaching it to perform a specific task [33,34]. This approach (apart from its accuracy) also has drawbacks, such as computational cost and training time, which in both cases is very high.

Depth estimation is a well-known topic of research, where very diverse studies can be found in a wide variety of projects including [35], which makes use of multi-camera arrays, those where sensors like LiDAR are used [36,37], or those that opt for the use of time-of-flight (TOF) devices [38,39]. The latter (the TOF-based projects) cannot be considered in our study due to the reflection effect generated by the nature of the materials of the targets to be manipulated as demonstrated in [3], mentioned in the introduction. The others present restrictions upon the illumination and the range.

The estimation of spatial coordinates based on the use of stereoscopic vision [40] is a widely used technique [41,42,43]. Algorithms can also be found that simulate it by using a single camera on a mobile platform [44], or by obtaining two slightly deviated images of a same scene at different points in time [45].

The use of RGB-D devices such as Kinect [46] or RealSense cameras [47], which provide not only color information but also depth information, bring another kind of solution when estimating spatial coordinates. However, they carry with them other problems such as the limitation of space (since they are devices of considerable dimensions), and the shadows/occlusions that they generate themselves (see Section 3.3). Methods such as [48,49] show the use of deep learning to infer depth from a single RGB image. Such methods need a large number of samples and are computationally very expensive during the training process.

**Figure 2:**
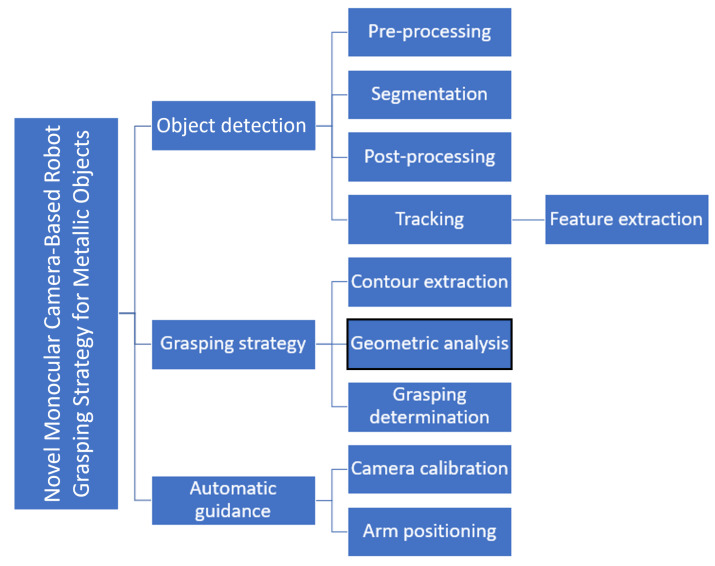
Schematic of the modules that make up the system.

In this paper, we propose a new solution for object detection and grasping determination in a challenging environment such as the CERN’s experimental facilities. For this purpose, we suggest using salient object detection for target detection, as well as a hybrid approach, combining image-based for physical features, and a novel geometrical analysis algorithm for grasping strategies (see Figure 2). In addition, for depth estimation and spatial coordinate determination, we propose to use an eye-in-hand system mounted on the end-effector of a robotic arm that simulates stereoscopic vision by capturing two frames from different positions by using the matrix of the robot, to balance the need for high accuracy with the constraints of the environment and equipment.

## 3. Materials and Methods

This chapter outlines the methods and equipment utilized in achieving the project’s goal. Section 3.1 focuses on object detection, presenting techniques for improving contrast to enable object detection in complex environments. Additionally, a novel technique for semantic segmentation is introduced, which allows for the identification of the most attractive object in the scene. This section also covers techniques for enhancing the accuracy and reliability of the segmented object, along with feature-based tracing methods for tracking the object during the positioning stage.

Moving on to Section 3.2, the contour-based grasping approach is explored as one of the primary methods for determining grasping points. A geometric analysis is performed to calculate important features that aid in the determination of the grasping points. These features, combined with mechanical conditions, define specific thresholds that are evaluated at antipode points along the contour, ultimately identifying the two best grasping points.

Section 3.3 addresses the environmental complexities that affect the methods of depth estimation during real interventions at CERN and the process of obtaining depth from a monocular camera. Detailed explanations are provided for subprocesses such as image calibration, disparity map calibration, and depth calibration.

Finally, Section 3.4 delves into the recreation of environmental conditions and equipment used during testing, as well as the performance achieved.

### 3.1. Object Detection

**Figure 3:**
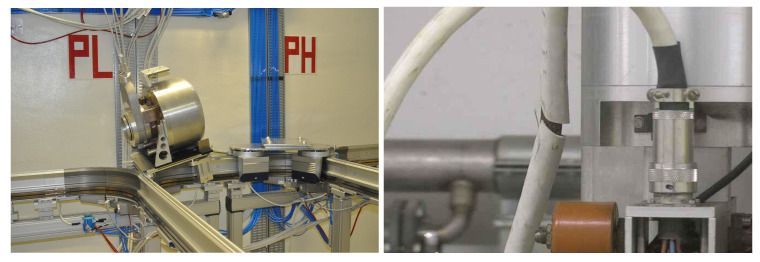
Real targets to be manipulated. (**left**) MEDICIS target out of the montrac (reach-ability test); (**right**) socket to be replaced.

Expert operators at CERN are faced with the challenge of handling delicate targets that require much care and attention, which can take a significant amount of time to complete. However, despite their expertise, there is always a risk of operator error or accidents that could damage the equipment or delicate objects. Additionally, recognizing these objects within a scene is complex due to the reflective properties of metallic materials and the resulting confusion in the detection process. Considering the object’s surface and potential shadows can improve the accuracy of object detection, which is important for ensuring the safety and success of the intervention. Figure 3 illustrates the delicate targets that expert operators at CERN must manipulate.

The following sections present a comprehensive approach to address the complexities involved in object detection. The techniques covered include preprocessing and advanced segmentation methods, as well as postprocessing techniques that can improve the accuracy and reliability of object detection.

#### 3.1.1. Preprocessing of the Image

**Figure 4:**
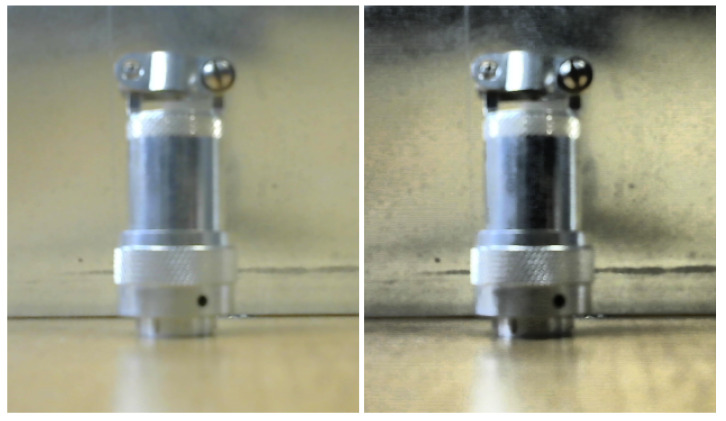
Classical socket used before and after the preprocessing. (**left**) original picture; (**right**) after applying CLAHE.

Histogram equalization is the most common technique for improving the contrast and balancing the brightness of a scene. Taking into account that the lack of contrast and the unstructured working environment are constant premises where lighting control is inaccessible, Adaptive Histogram Equalization (AHE) is presented as a better option than Global Histogram Equalization (GHE) when there is a wide range of luminance values. AHE performs piece-wise equalization of the image leading to a better constrast enhancement. Despite the improvements, this solution brings an increase in noise and may lead to over-amplification of contrast. To overcome these constraints, Contrast-Limited Adaptive Histogram Equalization (CLAHE) [50] has been used instead, which is a variant of AHE in terms of limiting the contrast amplification in order to reduce the noise amplification; see Figure 4.

The application of this technique facilitates the success of the task of object detection within a scene where the target and its environment share a featureless surface and a very low level of contrast where traditional methods cannot, as well as making it possible to deal with other cases such as partial occlusions or distinction between plain colors, which present an additional challenge.

#### 3.1.2. Segmentation of the Object and the Background

Robotic interventions at CERN take place in an unstructured environment, so it is not always possible to predict the specific types of objects to be handled. To address this challenge, this project utilizes deep learning salient object detection, a technique that is better suited to the problem than other conventional segmentation techniques (Table 1). The model used is U2-NET [51], which gives outstanding results compared to other salient object detection methods in datasets such as ECSSD [52]. The model segments accurately unknown objects of interest from the background within the uncertainty conditions. U2-NET is a two-level nested U-structure designed for SOD and it is capable of obtaining more contextual information in an image.

**Figure 5:**
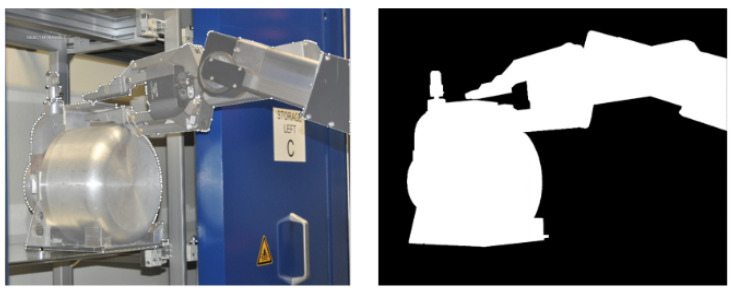
Labeling process: (**left**) segmentation by the hand of the interest object. (**right**) Mask after segmentation.

A dataset with relevant information, enabling the network to identify patterns that help to obtain high accuracy during the inference process, has been created by focusing only on metallic targets on surfaces of the same nature, taking images of real robotic interventions carried out within the CERN’s facilities. The goal is to accurately depict the desired concept in order to generate a higher acuity during pattern identification. In particular, the network’s ability to handle the shadows and brights in metallic objects in different lighting conditions was a key factor in its selection. In Figure 5, the labeling process of the dataset can be seen.

Then, by using data augmentation (see Table 2 with the specification/distribution used), where the original dataset was increased 15 times, achieving a set of 6880 images, and in order to prevent overfitting (that might influence the effectiveness of the trained model), we have additionally used the DUTS-TR [53], which is a dataset used for benchmarking the SOD algorithms.

**Figure 6:**
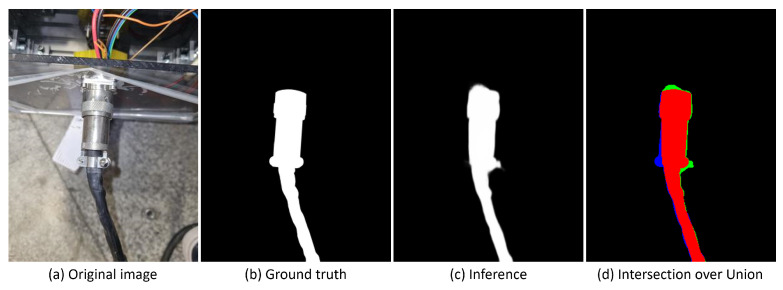
Intersection over union representation.

Intersection over Union (IoU) image segmentation has been used to evaluate the prediction accuracy of the model trained on our test dataset. Figure 6 shows the predicted mask, which is compared with the ground truth. Red coloring represents true positives, green coloring represents false positives, and blue coloring represents false negatives.

How these parameters are calculated is shown below:

- True Positives (TP) (1), area overlapped of both the Ground Truth (GT) and Segmentation Masks (SM).
  (1) TP=GT∧SM
- False Positives (FP) (2), the number of pixels predicted as part of the mask that does not match with the ground truth.
  (2) FP=(GT∨SM)−GT
- False Negative (FN) (3), part of the ground truth not predicted on the predicted mask.
  (3) FN=(GT∨SM)−SM

Equation (4) evaluates the level of overlap between both masks (ground truth and inferred) at the pixel level.

(4) $$IoU=\frac{TP}{TP+FP+FN}$$

After establishing the evaluation method, the training process was carried out using the following configurations:

1. Original pre-trained model;
2. Model trained with DUTS-TR and own metallic objects dataset and data augmentation.

**Figure 7:**
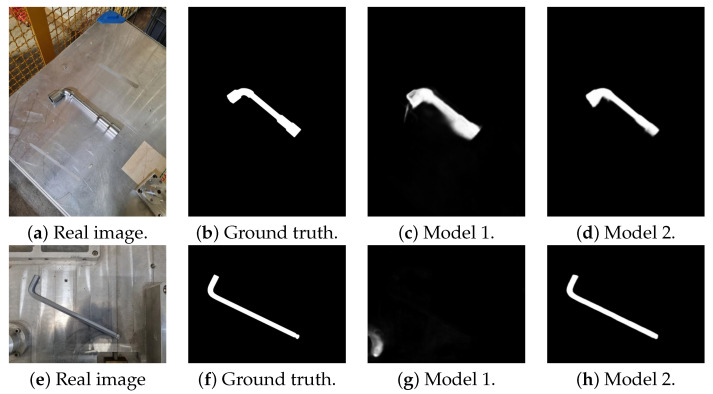
Qualitative comparison between original pre-trained model (Model 1) and model trained with DUTS-TR dataset and metallic objects dataset (Model 2).

The modified model improves object detection in challenging metallic environments by producing smoother edges and better-filled internal parts, as indicated in the qualitative results (see Figure 7). It also excels at handling reflections, shadows, and low contrast, leading to enhanced performance in generating the final object mask during postprocessing.

The analysis was concluded by calculating the intersection over union (IoU) for the test images in both cases, which comprised 20% of the complete dataset. Subsequently, the mean and standard deviation of the IoU were determined. The quantitative outcomes are presented below.

Based on the results presented in Table 3, configuration 2, which involves training a model with the DUTS-TR and own metallic objects datasets and applying data augmentation, has been selected for the remainder of the project. Model 2 outperformed the other configuration by demonstrating a 59% improvement in performance and a 26% reduction in data variance in inferring metallic objects.

#### 3.1.3. Postprocessing of the Mask

With the aim of obtaining a reliable and accurate enough mask to facilitate the object detection process, classical computer vision techniques have been used to mitigate the small defects that may appear during the inference process, as listed below:

- Threshold of the inferred image, using Otsu method [54]: Thanks to both its simplicity and its effectiveness in isolating an object of interest from its surroundings, Otsu automatically finds the best threshold value by returning high-quality binarized images.
- Mask refinement: To finish refining the obtained masks, it is required to fill in the incomplete parts of the area of the target, and erase those clusters of pixels not belonging to the object of interest by sequentially applying the erosion and dilation techniques, thus transforming the images by means of the techniques known as opening and closing.

Finally, a Gaussian filter was applied in order to blur the contours and edges of the segmented target, thus softening the contrast between the environment and the object.

#### 3.1.4. Tracking Object

To facilitate the operator’s task, and once the target is detected, the operator is given the possibility to track it during the teleoperation process as an auxiliary functionality (without interfering with the results obtained) of the algorithm. This derives from the work in [55], after a thorough review of the current status (see Table 4) of the following tracking algorithms implemented by OpenCV:

GOTURN [56] has not been taken into account due to the fact it is based on a CNN, so the performance will be related to the dataset used for training the model, and this is not the aim of the project.

For recovering the tracking once the tracker is not capable of retrieving by itself, we decided to use SIFT since it is the algorithm that has shown the best results (among others such as SURF, Oriented fast and Rotation Brief (ORB) [57], and Binary Robust Independent Elementary Features (BRIEF) [58]) when dealing with metallic pieces on metallic backgrounds.

### 3.2. Contour-Based Grasping

The target contour extraction is the main pillar of the grasping point determination task, hence the accuracy of this step will determine the success of the whole task. Therefore, after the mask is inferred, the contour of the target must be extracted therefrom. For this matter, we have used an OpenCV method (*cv.FindContours*) which returns an array with the contours of every single closed polygon. Since the mask is from a Region of Interest (ROI), we just consider the largest one as the interest contour. In addition, the method has a flag to choose the approximation, allowing one to choose the number of points stored per contour. In this project two of four have been used:

- CHAIN_APPROX_NONE, maintains all the points of which a contour is composed, i.e., the maximum absolute difference between two X and Y coordinates is 1.
- CHAIN_APPROX_SIMPLE, uses the compression of vertical, horizontal, and diagonal segments while maintaining their endpoints; a rectangular shape would be represented by just four points.

**Figure 8:**
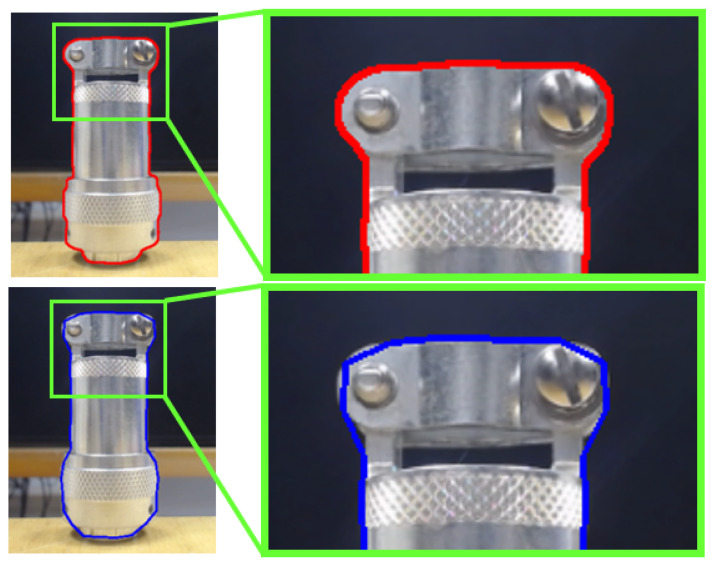
Contour extraction methods. Top-image method 1: CHAIN_APPROX_NONE; bottom-image method 2: CHAIN_APPROX_SIMPLE.

Figure 8 shows the difference between two contours’ approximations from the same target by using each of the methods. The first method, in spite of carrying a greater computational load, will be critical in the result. Even so, its use considerably reduces the number of points to be treated, greatly alleviating the processing time in cases of excessively complex contours.

#### 3.2.1. Geometric Analysis

A set of mathematical quantities known as raw moments are usually used to calculate features such as the centroid, size, or orientation of a target within an ROI. These quantities can be calculated at the pixel level and represented as a set of numerical values, which are used to determine the best possible grasping points, thanks to the information provided by encoding the characteristics of the object listed below:

(5) $$M_{ij}=\;\sum_{x}\sum_{y}x^{i}y^{j}I\left(x,y\right)$$

**Figure 9:**
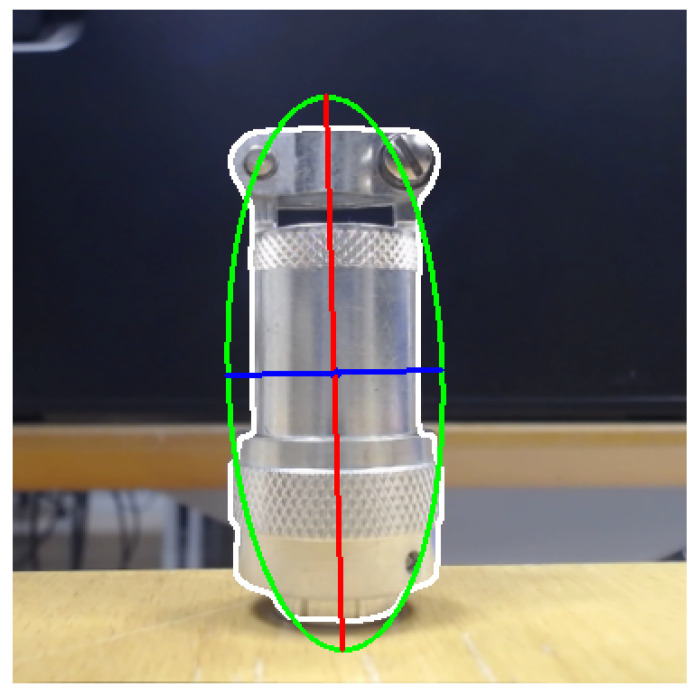
Inertia axes are represented in the image and the ellipse that best fits the object contour.

- Area (A): based on Equation (5), where *I(x,y)* represents the intensity of a pixel in a grayscale image, the area of a target can be calculated for a moment $M_{00}$ (see Equation (6)):
  (6) $$A=M_{00}=\sum_{x}\sum_{y}x^{0}y^{0}I\left(x,y\right)$$
- Gravity center: for the same moment ($M_{00}$) location of the center of mass can be calculated by using Equation (7).
  (7) $$Centroid\left\{\bar{x},\bar{y}\right\}\;=\;\left\{\frac{M_{10}}{A},\frac{M_{01}}{A}\right\}=\;\left\{\frac{M_{10}}{M_{00}},\frac{M_{01}}{M_{00}}\right\}$$
- Central moments: unlike the raw moments, the central moments are invariant to the translations (localization) of the target. To do this, it extracts the centroid from X and Y (see Equation (8)).
  (8) $$\mu_{ij}=\;\sum_{x}\sum_{y}{\left(x-\bar{x}\right)}^{i}{\left(y-\bar{y}\right)}^{j}I\left(x,y\right)$$
  In addition, some interesting features such as inertia axis, orientation, and eccentricity can be derived from the spatial moments by using the calculated central moments up to the second order (see Equation (9))
  (9) $$\mu_{ij}=\frac{M_{ij}}{M_{00}}-{\left(\frac{M_{10}}{M_{00}}\right)}^{i}*{\left(\frac{M_{01}}{M_{00}}\right)}^{j}$$
- Inertia axis: the rotational axis with maximal or minimal inertia. They can be represented with an ellipse, which must fit perfectly the target’s contour, by calculating their module as follows.
  (10) $$Imod_{min}={\left(\frac{4}{\pi }\right)}^{1/4}*{\left(\frac{inertia_{max}^{3}}{inertia_{min}}\right)}^{1/8}$$
  (11) $$Imod_{max}={\left(\frac{4}{\pi }\right)}^{1/4}*{\left(\frac{inertia_{min}^{3}}{inertia_{max}}\right)}^{1/8}$$
- Orientation: the object rotation along the Z-axis in the camera coordinates (see Equation (14)). Taking into account Figure 9, the rotation can be calculated using the minimum inertia axis (see Equation (13)), which was estimated for the eigenvalues (see Equation (12)).
  (12) $$cov\left[I(x,y)\right]=\left[\begin{array}{cc} \mu_{20} & \mu_{11}\\ \mu_{11} & \mu_{02} \end{array}\right]$$
  (13) $$\lambda_{1}=\frac{\mu_{20}+\mu_{02}}{2}-\frac{\sqrt{4\mu_{11}^{2}+{\left(\mu_{20}-\mu_{02}\right)}^{2}}}{2}$$
  (14) $$\theta =\frac{1}{2}arctan\left(\frac{2\mu_{11}}{\mu_{20}-\mu_{02}}\right)$$
- Roundness and Eccentricity: Although they are very similar, they are calculated differently and have different uses. We focus on eccentricity, which is widely used for comparison, as it is a very reliable reference point. It is calculated as shown in Equation (15).
  (15) $$\varepsilon =\frac{{\left(\mu_{20}-\mu_{02}\right)}^{2}-4\mu_{11}^{2}}{{\left(\mu_{20}+\mu_{02}\right)}^{2}}$$

**Figure 10:**
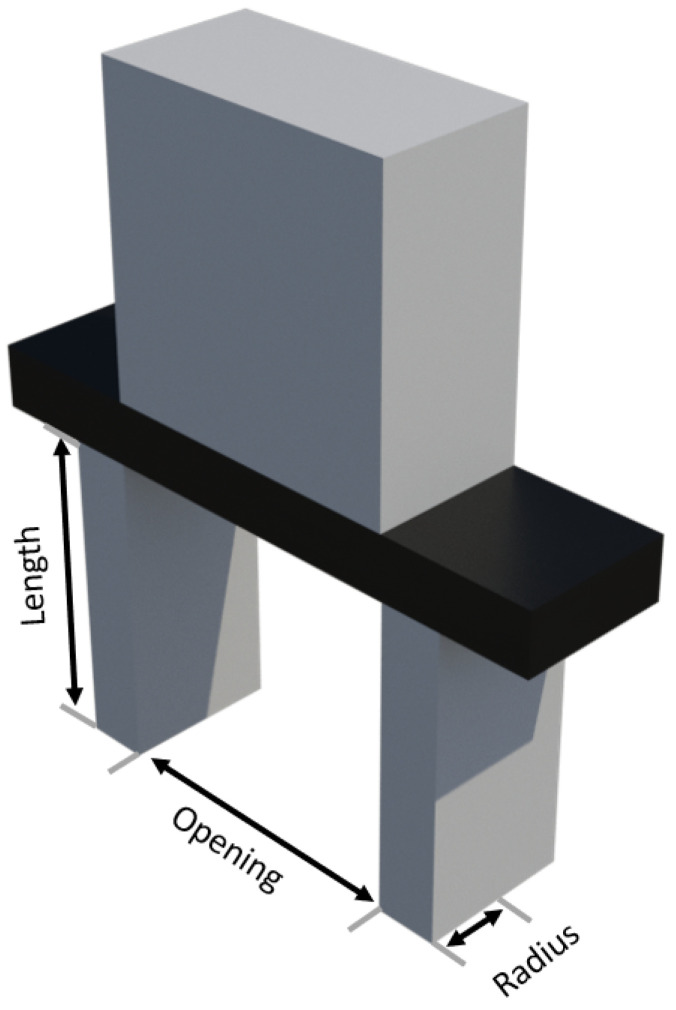
Characterization of the gripper geometry.

Now that both contour and geometrical analysis have been completed, it is necessary to consider the gripper geometry, which is depicted in Figure 10, with the aim of studying the suitable mechanical properties-based grasping strategies to guarantee reliable stability and slip-free grasping.

#### 3.2.2. Maximum Curvature or Surface Uniformity Threshold

The aim of this work is to provide in an analytical way two grasping points to allow the operator either to execute the task in a teleoperated or guided way. For such an effect, the quality of these points has to guarantee stability and reliability to avoid slippage. Since the contour evaluated in the steps above is composed of a bundle of pixels, locating two opposite points across the plane that guarantee the required parallelism of the gripper becomes a non-trivial task.

Algorithm 1 has been developed to overcome this difficulty by extracting from the object contour the diametrically opposite points of its surface that should come into contact with the gripper (of radius r), where the adjacent area is sufficiently smooth to provide a suitable grip.
**Algorithm 1** Maximum curvature or surface uniformity threshold      **Input:** Tangents, Object Contour, Eccentricity 1:  Calculates the points of the tangent line of 2 times the radius of the length of the gripper with the grasping point in the center. 2:  Calculates the percentage of the line that is touching the contour of the object. 3:  **if** eccentricity is below a roundness threshold **then** 4:      a low uniformity threshold is set. 5:  **else** 6:      a high uniformity threshold is set. 7:  **end if** 8:  **if** no points of the line are inside the contour **then** 9:      **if** the percentage of the line is above the uniformity threshold **then**10:          **Output:** valid grasping point.11:      **else**12:          **Output:** invalid grasping point.13:      **end if**14:  **else**15:      **Output:** invalid grasping point.16:  **end if**

This algorithm allows for determining if the surface is flat enough to ensure a good percentage of contact between the object with the parallel fingers of the robot’s gripper. However, in cases where the object’s shape is highly complex, ensuring a high percentage of surface contact may not be feasible. In such situations, it may be possible to modify the maximum curvature or surface uniformity threshold. This threshold can be adjusted by the operator as needed, taking into account the complexity of the object at hand and leveraging the operator’s experience. In addition, it considers the chance of grasping both round and low eccentricity objects, which present less uniformity and necessitate different approach strategies.

#### 3.2.3. Slip Threshold Defined by Gripper Material and Object

Based on Coulomb’s law of friction established in 1781, and the friction coefficient shown in Table 5 for some of the most commonly used materials in metallic objects, we can predict the forces needed for holding or translating an object to avoid slippage.

Nevertheless, focusing on the geometrical point of view, we can notice that the total applied force generates an angle θ (see Equation (16), μ is the coefficient of static friction), where the set of its resultant vectors will generate an angle with the normal which is known as the *friction cone*, and its θ will be the friction coefficient.

(16) θ=arctan(μ)

**Figure 11:**
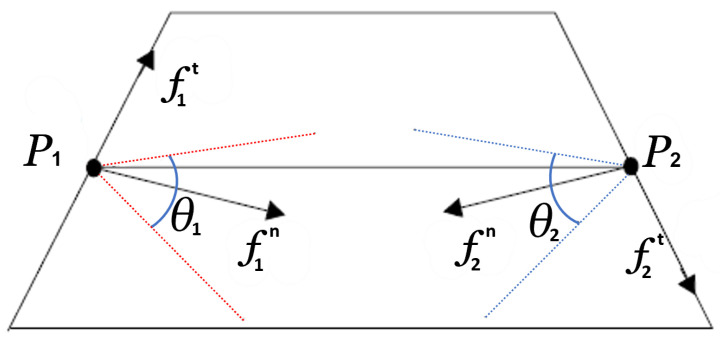
Geometrical explanation of stable grasping points (P1 and P2 with their respective frictions cones in red and blue) where $f^{t}$s are the tangential forces, $f^{n}$s are the normal forces, and θs are the angles of the friction coefficients. Enhanced image from [28].

Then, it follows that the segment that intersects the two grasping points must be included between friction cones derived from each point (see Figure 11) to grant a stable grasp.

#### 3.2.4. Parallelism between Grasping Points

**Figure 12:**
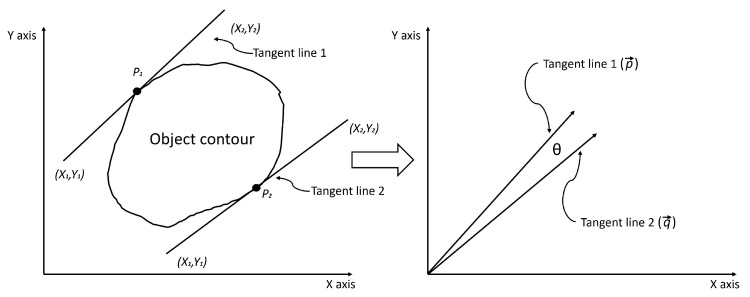
Representation of grasping tangent lines into vectors.

The use of the tangents generated by each of the points determined in the previous step allows us to study their parallelism. These tangents are obtained by subtracting the initial (x1, y1) and final (x2, y2) coordinates of each tangent and thus produce a vector representation (see Figure 12).

Then, performing the scalar product of both vectors will give us the angle (θ) they form by applying the equation below:

(17) $$\theta =\arccos\left(\frac{\vec{p}\cdot \vec{q}}{\left|\vec{p}\right|\left|\vec{q}\right|}\right)$$

This θ will indicate the level of parallelism of the two points, where 0 will be fully parallel.

#### 3.2.5. Distance from a Point to a Line as a Threshold

**Figure 13:**
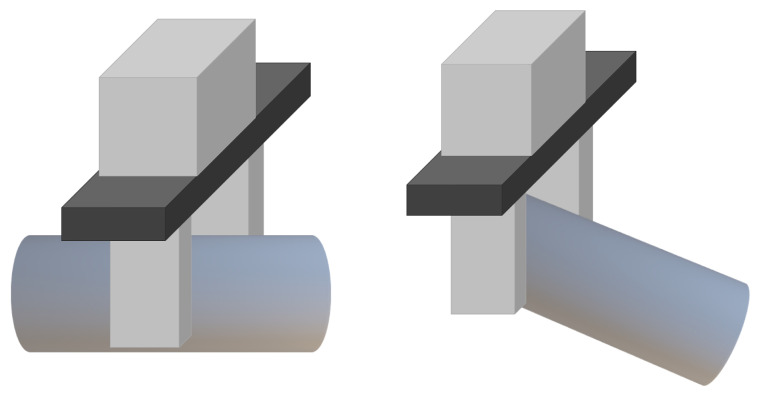
Representation of good (**left**) and bad (**right**) grasping in terms of the instability produced if the grasping points are close or far from the object´s centroid.

An extra layer to guarantee both the effectiveness and stability of the grasping (see Figure 13) will be by applying the well-known equation from a point to a line (18), where our line is represented as the union between the grasping points $P_{1}$ ($x_{1}$,$y_{1}$) and $P_{2}$ ($x_{2}$,$y_{2}$), and the point is the centroid of the target ($x_{c}$,$y_{c}$).

(18) $$Distance\left(P_{1},P_{2},\left(x_{c},y_{c}\right)\right)=\frac{\left|\left(x_{2}-x_{1}\right)\left(y_{1}-y_{c}\right)-\left(x_{1}-x_{c}\right)\left(y_{2}-y_{1}\right)\right|}{\sqrt{{\left(x_{2}-x_{1}\right)}^{2}+{\left(y_{2}-y_{1}\right)}^{2}}}$$

The threshold has to be tuned manually since the stability factor determined by the *Distance* will be influenced by the type of targets to be handled. Again, the experience of the operator makes a difference in the performance within the scope.

### 3.3. Monocular Depth Acquisition

**Figure 14:**
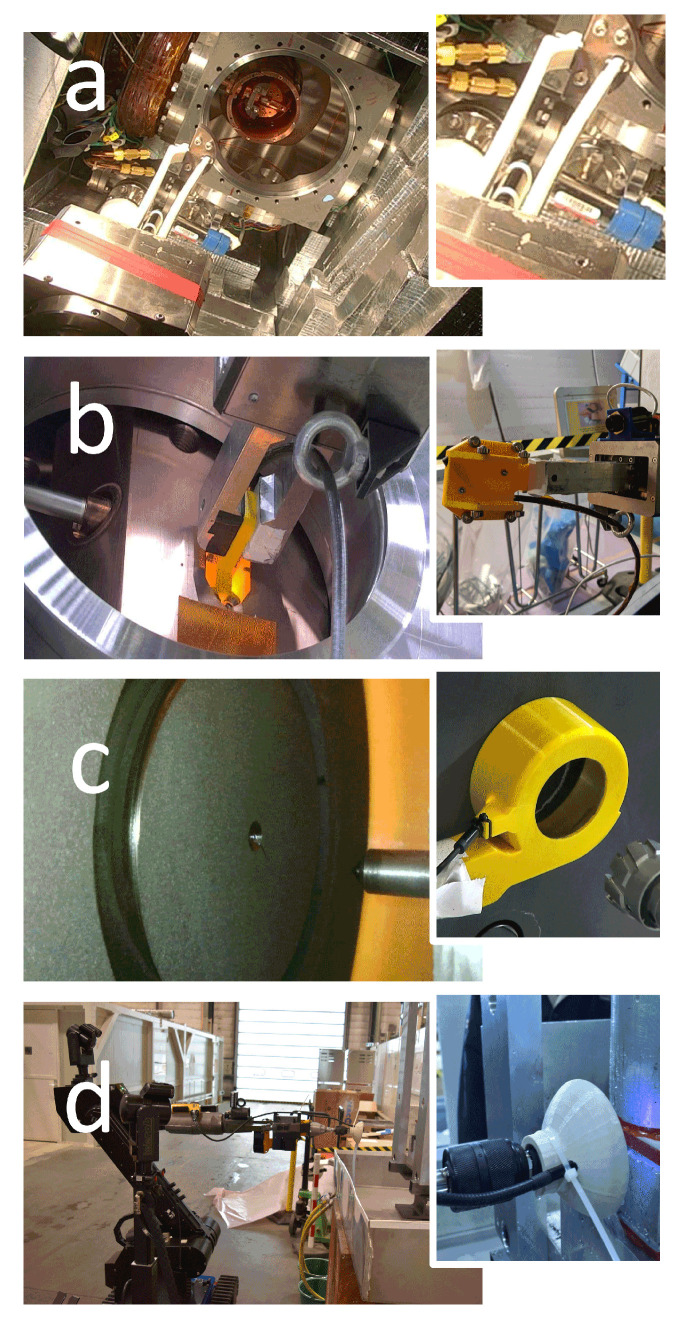
Small representation of some of the tools (used in real interventions) intended to be used with endoscopic cameras mounted in (**a**) the middle of the fingers; (**b**) inside of inspection trolley; (**c**) inside a coiled vacuum cleaner; (**d**) inside a driller and water drainer.

In certain situations, the use of endoscopic cameras is necessary due to space constraints (see Figure 14). However, these cameras often have limited imaging capabilities and can produce low-quality images that are difficult to interpret. Additionally, there is a lack of confidence in time-of-flight devices, which rely on the properties of the target material to operate effectively. As a result, these devices may produce inaccurate or unreliable results in certain scenarios.

**Figure 15:**
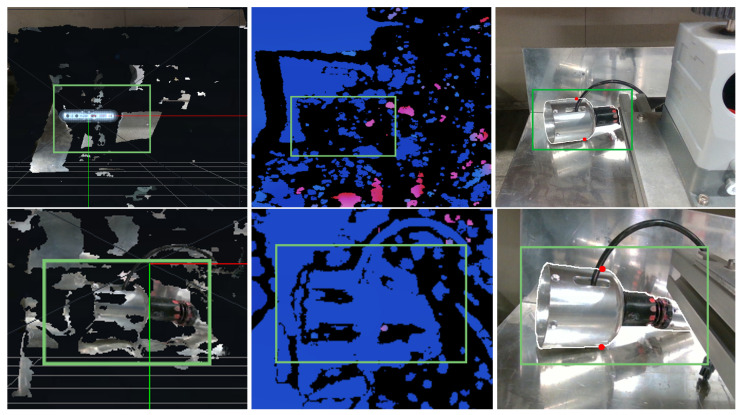
Graphical representation of our system against RGB-D cameras (first row RealSense D415, second row RealSense D405): left image shows the 3D reconstruction, in green the ROI where the target is actually located; center image shows the deep information; right image shows the contour and grasping points estimated by our solution.

Moreover, our tests have shown that the use of RGB-D cameras, such as RealSense (Intel Corporation, Santa Clara, CA, USA), does not always provide sufficient confidence for their use. One issue with these cameras is that their infrared (IR) light mesh and stereo pair can fall into partial occlusions, which can cause the target object to be obscured and disappear from the disparity map or depth map (see Figure 15). This can make it challenging to obtain accurate measurements and understand the shape and structure of the object being imaged.

To address these challenges, additional algorithms are often necessary to fix or fill the IR shadow regions that result from partial occlusions. For example, in [60], an object is assumed to have a similar color over the entire surface and a similar depth of adjacent pixels to fill the unmeasured areas. This approach can be effective in certain cases, but it may not always provide accurate results. To overcome the issue of shadow regions in monocular cameras, we propose an adjustable baseline distance between the two pictures taken. This provides a solution in certain ranges, allowing the user or operator, after calibration, to try different distances and qualitatively judge which provides the best results.

#### 3.3.1. Stereo Vision with a Monocular Camera

Stereo vision using a monocular camera involves taking two pictures with a known baseline, allowing for the creation of a disparity map that correlates pixel differences between stereo images with distance. This map is represented as a two-dimensional Float32 matrix, where lower values correspond to greater distances and higher values to closer objects. To establish the relationship between matrix elements and actual depth, calibration is divided into three parts.

#### 3.3.2. Images for Calibration

**Figure 16:**
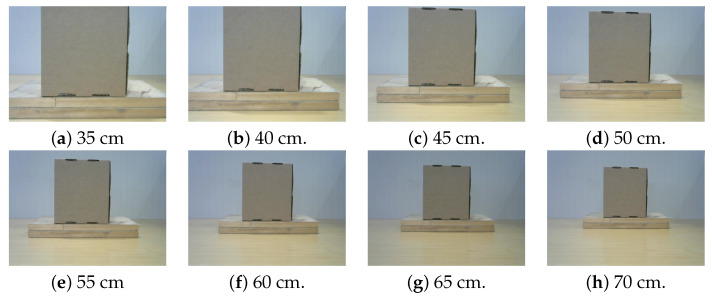
Calibration images taken at different distances from 35 to 70 cm with a baseline of 3 cm.

Apart from the well-known calibration based on Zhang’s method [61] used for the extraction of both the intrinsic and extrinsic parameters of the camera, it is necessary to calibrate the disparity map and determine object distance accurately, plain objects such as boxes should be photographed at various distances, typically in the range of 30–70 cm. The selection of this range is influenced by the reach of the robotic arm and the appearance of the object from the camera’s perspective. The baseline, or the difference between the two pictures, must also be considered during calibration. For this study, baseline values of 2, 3, and 4 cm were utilized. Sample calibration images are depicted in Figure 16.

#### 3.3.3. Disparity Map Calibration

Disparity estimation algorithms can be local or global. Local methods evaluate each pixel independently while global methods consider the whole image, but are more computationally intensive. A hybrid approach, semi-global matching, combines both methods. However, it still requires significant computation time. The Semi-Global Block Matching (SGBM) algorithm addresses this issue by computing disparity using a smaller block of pixels. SGBM uses block-based cost matching that is smoothed using path-wise data from multiple directions [62]. Rectified left and right stereo images serve as input for SGBM. The rectification process aligns the vertical coordinates of corresponding pixels in both images so that epipolar lines are parallel to the horizontal axis [63].

**Figure 17:**
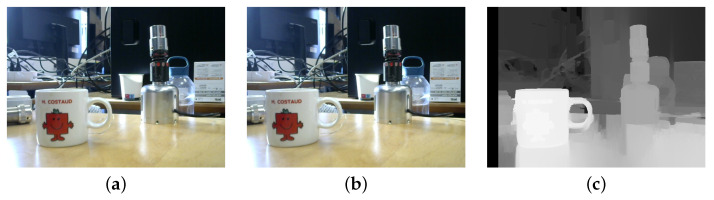
The overall process of disparity. (**a**) Left image; (**b**) right image; (**c**) disparity map.

The SGBM matcher can produce inaccurate disparity maps due to texture-less areas, occlusions, and depth discontinuities. To solve these problems, a filtering technique is applied to align the edges and propagate disparity values from low-confidence regions. For this, we already have a left matcher (SGBM matcher) and we create a right matcher by passing the stereo images from right to left. Then, both matchers are used to compute disparity maps, which are then passed to the filter with the source left view. This improves accuracy by reducing errors (see Figure 17).

The quality of the resulting disparity map is reliant on the matching accuracy. Higher resolution cameras can capture more detailed information, enabling more accurate matching between corresponding images. As a result, lower resolution cameras may not capture sufficient scene details, leading to incorrect or noisy matching and lower overall quality in the resulting disparity map. This project utilized a camera resolution of 640x480 to capture the scene and calculate its depth through disparity maps.

#### 3.3.4. Depth Calibration Based on Intensity

**Figure 18:**
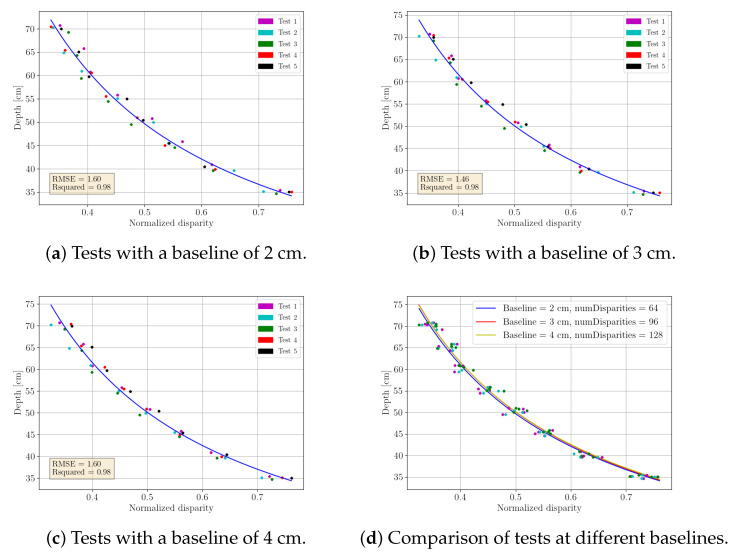
Calibration curve of normalized disparity against depth for different baselines.

The disparity map alone does not provide depth information, so images from Section 3.3.2 are used to relate pixel values to distance. The distance is measured using a laser meter and the relation between distance and the pixel value is determined using the “curve fit” method from SciPy library [64], which fits the data to a predefined function. Tests were conducted with baselines of 2, 3, and 4 cm (see Figure 18), with only one parameter (number of disparities) being changed in each test. It was observed that this parameter changes linearly, increasing by 32 for every 1 cm of change in baseline, resulting in values of 64, 96, and 128 for baselines of 2, 3, and 4 cm, respectively. Therefore, it is feasible to compute all the values within that range for this parameter.

### 3.4. Test Setup and Performance

In this study, the implementation of the algorithm was divided into two parts: the training of the neural network architecture and the application for grasping points calculation. The training of the neural network architecture required a high computational cost, which made it impossible to be performed on a typical computer. Hence, the training was executed on a CERN server with a processor Intel(R) Xeon(R) Silver 4216 CPU @ 2.10 GHz (Intel Corporation, Santa Clara, CA, USA), a GPU NVIDIA Tesla V100S 32 MB (Nvidia Corporation, Santa Clara, CA, USA), and 32 GB RAM, which resulted in a more efficient and rapid completion of the training process, taking approximately 150 h.

To further evaluate the algorithm’s performance, the application for grasping points calculation was tested on a laptop with a processor Intel(R) Core(TM) i5-9300H CPU @ 2.40 GHz, a GPU Nvidia Geforce GTX 1650 4 MB, and 12 GB RAM. This allowed for a better understanding of the algorithm’s behavior when integrated into a portable device, a crucial aspect of its practical applications. Additionally, a series of tests were conducted to measure the performance of the algorithm with various combinations of features. The results of these tests, summarized in a Table 6, indicated that the object segmentation component of the algorithm consumed the majority of the execution time due to its intensive computational requirements.

**Figure 19:**
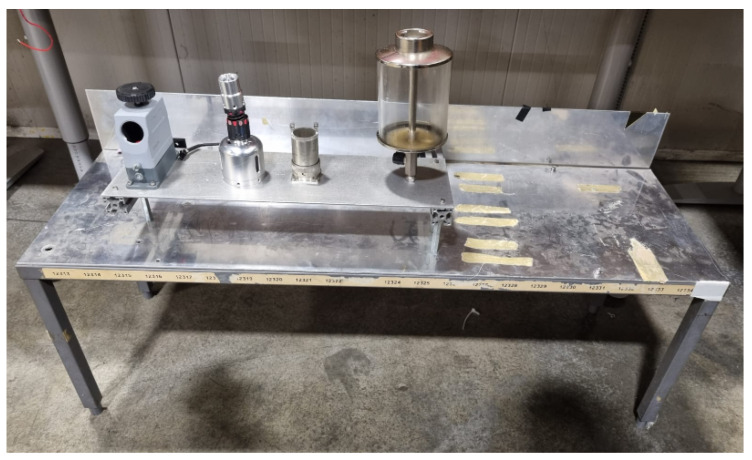
Test bench used for testing the algorithm.

The performance of our algorithm was evaluated by simulating real-world conditions using a specialized test bench. The bench consisted of a metallic surface with objects that created shadows and reflections, adding difficulty to the detection. This testing method ensured accurate results for evaluating the effectiveness and reliability of the algorithm. The test bench and conditions are depicted in Figure 19.

The selection of equipment and devices was a crucial factor in the project as they were required to execute the algorithm. The devices were procured from CERN laboratory and their features are described in Table 7. The robotic arm and camera used were designed to function independently.

## 4. Results

In this chapter, we present the results of two tests that were conducted to evaluate the effectiveness and reliability of the algorithm in performing its intended task. The primary objectives of these tests were to determine the success rate of the algorithm and to assess the level of error in its output. Through a detailed analysis of the results obtained from these tests, we gained a deeper understanding of the algorithm’s performance and its ability to deliver accurate and reliable results in the specific application it was designed for.

### 4.1. Repeatability of Grasping Points from Different Joint Configurations

**Figure 20:**
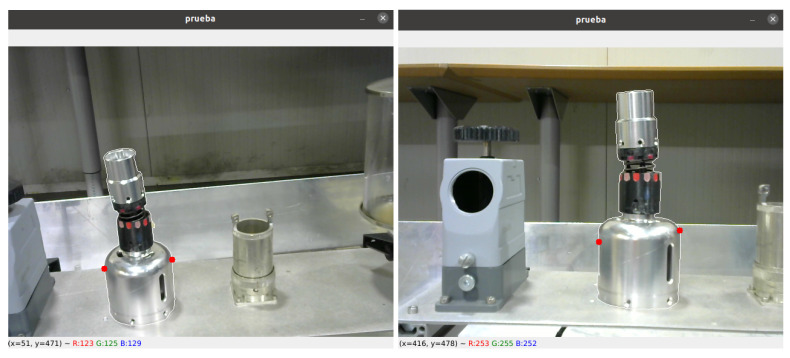
Determination of the grasping points of an element from various perspectives to assess the repeatability of the algorithm.

We conducted two tests to evaluate the repeatability of the algorithm. The object of interest was placed in a specific position on the test bench and a robotic arm equipped with a camera mounted on the end-effector was used to run the algorithm at different joint configurations. The purpose of these tests was to observe variations in the *x*, *y*, and *z* coordinates in the robot frame to determine the algorithm’s accuracy and consistency. Figure 20 shows the two tests and illustrates the object segmented with a white border and the grasping points marked with red dots. The exact location of the grasping points may vary slightly due to the calculation being based on the object’s contour, potentially impacting the final results of the tests.

**Figure 21:**
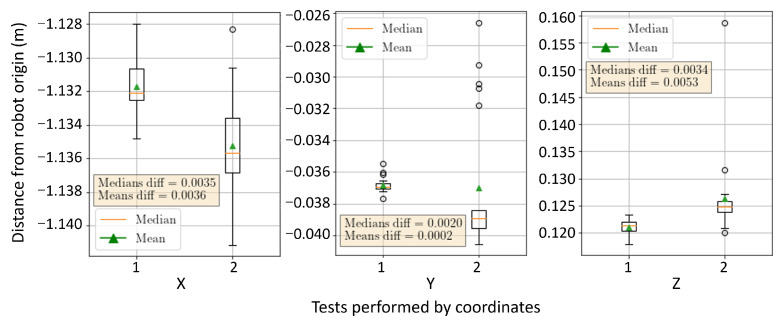
Results for spatial conditions after two tests in different joint configurations.

Figure 21 shows larger dispersion in the x-axis (1 cm deviation) than in the y-axis and z-axis (measured in mm), suggesting possible outliers or other variables. Mean and median values of the scatter points are close to each other, indicating a symmetrical distribution.

### 4.2. Algorithm Distance Measurement Compared with a Tested Laser Meter as Benchmark

**Figure 22:**
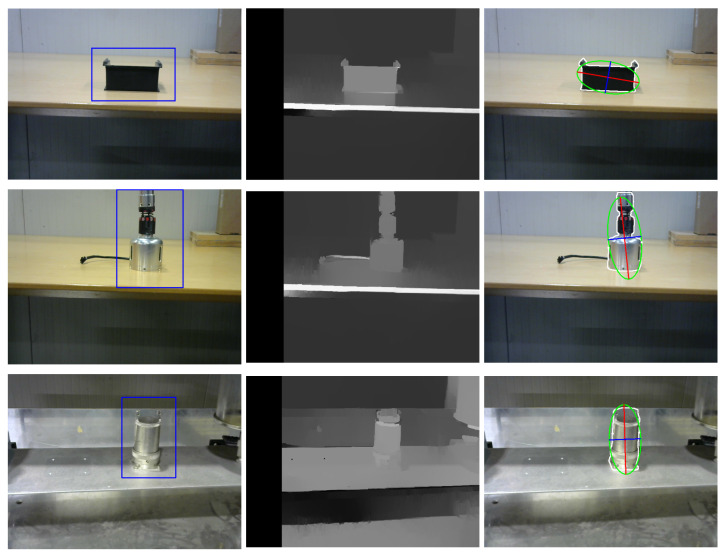
Three different tests were performed to obtain the accuracy of the algorithm.

We conducted three tests to assess the accuracy of the algorithm. The robotic arm was equipped with a camera and laser meter, and three objects were placed in specific positions. The absolute difference between the mean distances calculated by the laser meter and algorithm were measured to determine accuracy. Figure 22 shows the three different tests conducted under varying environmental conditions.

**Figure 23:**
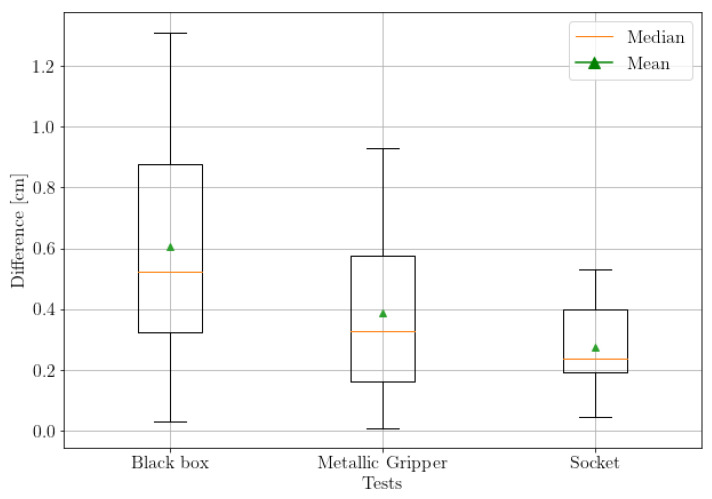
Difference obtained between the distance obtained by the benchmark and the algorithm.

The box plot in Figure 23 clearly illustrates the accuracy achieved in each test and the distribution of the scatter data. The results indicate that the objects for which the algorithm was specifically designed yielded promising results, with an error of less than 1 cm. Among these objects, the socket exhibited the minimum difference error, while the maximum difference error was observed in the case of the black box, approaching 1.3 cm. Moreover, the surface of the object being detected appears to have a significant impact on the algorithm’s performance. In this study, the socket, which has the least shiny surface among all the objects, produced the best results. Additionally, the means and median values for all tests are close to each other and less than 0.6 cm, indicating a symmetrical distribution of data with a high level of precision.

## 5. Discussion

### Future Work

This work not only shows the complexity to front metallic targets over metallic environments, but also the challenge of dealing with primarily unfamiliar objects. For these matters, the application of techniques such as few-shot learning [65,66] or one-shot learning [67] may be useful due to their ability to handle limited data and unseen classes. These solutions would allow for removal of the feature extractor algorithms from the equation for tracking recovery, in addition to enhancing the trained model for this work as tests and interventions are being carried out.

Furthermore, Self-supervised Learning [68,69], which uses unsupervised methods to learn features from data, may be useful to enhance the model too, creating a more and more robust solution according to the results shown on this project.

Although the system is fully operational and integrated into the CERNTAURO Framework [70], it eventually must be integrated into the CERN Robotic Graphical User Interface (GUI) [71] to be used in real scenarios, since it has been already used during preparation tests for interventions, showing its high level of accuracy and reliability.

Further work on sensor fusion for grasping determination beyond vision is planned, by using the benefits of already available sensors in the robot and end-effector tools, such as force–torque and tactile sensors [72].

## Figures and Tables

**Table 1:** Qualitative comparison of different segmentation methods after CLAHE application.

Segmentation Methods	Limitations	Evaluation
Thresholding	Prone to losing important object details and detecting background as part of the object. Sensitive to variations in brightness and shadows.	Not suitable
Region-Based	Complex to implement and may not be universally applicable. Sensitive to variations in brightness and shadows.	Not suitable
Edge/Boundary-Based	Requires high contrast between the object and background, and may fail if contrast is not sufficient. Sensitive to variations in brightness and shadows.	Not suitable
Neural Networks	Produces excellent results for known objects, but not appropriate for unknown targets	Not suitable
Deep Learning (SOD)	Produces exceptional results when performed in a region of interest, rather than the entire image. Does not require prior knowledge of the object.	Suitable

**Table 2:** Transformations, probabilities, and limits used in the data augmentation.

N°	Transformation	Probabilities	Limits
b	Horizontal Flip	0.5	N.A.
c	Vertical Flip	0.2	N.A.
d	Scale	0.5	min = Size, max = Size × 1.2
e	Rotate	0.5	min = 30, max = 30
f	RGB shift	0.5	R = 25, G = 25, B = 25
g	Blur	0.5	B = 1
h	Brightness	0.5	min = B − 0.2, max = B + 0.2
i	Contrast	0.5	min = C − 0.3, max = C + 0.3
j	Saturation	0.5	min = S − 0.2, max = S + 0.2

**Table 3:** Quantitative comparison between the inference using the original and the re-trained model.

Training	Mean	Standard Deviation
1	0.511	0.245
2	0.814	0.182

**Table 4:** Qualitative tests observed using different trackers.

	Tracking Accuracy	Ability to Retrieve Tracking	Management of Partial Occlusions	Stop Tracking When Object Is Lost	Frames per Second
Boosting	Good	NO	YES	NO	40
MIL	Good	NO	YES	NO	25
KCF	Good	YES	YES	YES	90
TLD	Bad	NO	NO	NO	70
MedianFlow	Good	NO	YES	YES	1000
MOSSE	Regular	YES	YES	YES	1000
CSRT	Good	NO	YES	NO	35

**Table 5:** Coefficient of friction for some metallic materials (fragment) [59].

Materials and Material Combinations		Surface Conditions	μ Static
Aluminum	Aluminum	Clean and Dry	1.05–1.35
Aluminum	Aluminum	Lubricated and Greasy	0.3
Aluminum–bronze	Steel	Clean and Dry	0.45
Aluminum	Mild Steel	Clean and Dry	0.61
Steel	Steel	Clean and Dry	0.5–0.8
Steel	Steel	Lubricated and Greasy	0.16
Wood	Clean Metal	Clean and Dry	0.2–0.6
Wood	Wet Metals	Clean and Dry	0.2
Silver	Silver	Clean and Dry	1.4
Silver	Silver	Lubricated and Greasy	0.55

**Table 6:** Performance achieved in every module of the algorithm.

Module	Time (ms)
Tracking	20
Preprocessing	1
Object Segmentation	80
Geometrical Analysis	5
Grasping Determination	30
Image Capture	10
Depth Calculation	5

**Table 7:** Description of the devices used during the tests.

Image	Model	Description
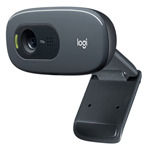	C270 HD Webcam	Max Resolution: 720 p/30 fps Camera megapixel: 0.9 Focus type: fixed focus Lens type: plastic Built-in mic: Mono Mic range: Up to 1 m Diagonal field of view (dFoV): 55°
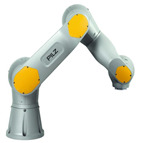	PRBT 6	Payload: 6 kg load Operating range: 741 mm Degrees of freedom: 6 axes Repetition accuracy position: 0.2 mm Weight: 19 kg Supply: 24 VDC
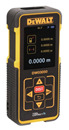	DW03050-XJ	Index of protection: IP54 Range: 50 m Precision: +/−1.5 mm

## Data Availability

The data used in this study contain sensitive information regarding the processes at CERN, and as such, cannot be shared publicly due to privacy concerns. Nevertheless, researchers who seek to access this data may request access through the corresponding author. Requests will be evaluated on a case-by-case basis. While we recognize the importance of data sharing and transparency in research, protecting the privacy and confidentiality of our study participants remains our top priority. Therefore, we are committed to taking all necessary measures to ensure the ethical use of data and safeguarding the privacy of our participants.

## Abbreviations

The following abbreviations are used in this manuscript:

ToF	Time of Flifht
MSM-8	Master-Slave Manipulator Mk. 8
AI	Artificial Intelligence
HOG	Histograms of Oriented Gradients
SIFT	Scale Invariant Feature Transform
SURF	Speed-Up Robust Features
ML	Machine Learning
DL	Deep Learning
CNN	Convolutional Neural Networks
R-CNN	Region Based Convolutional Neural Networks
RL	Reinforcement Learning
RGB	Read Green Blue
RGB-D	Read Green Blue Depth
CERN	European Organization for Nuclear Research
CDF	Cumulative Distribution Function
AHE	Adaptive Histogram Equalization
GHE	Global Histogram Equalization
CLAHE	Contrast-Limited Adaptive Histogram Equalization
SOD	Salient Object Detection
IoU	Intersection over Union
TP	True positives
GT	Ground Truth
SM	Segmentation Masks
FP	False Positives
FN	False Negative
ORB	Oriented fast and Rotation Brief
BRIEF	Binary Robust Independent Elementary Features
ROI	Region of Interest
IR	InfraRed
2D	2 Dimensions
3D	3 Dimensions
SGBM	Semi-Global Block Matching
GUI	Graphical User Interface
